# Development of a photochemical thrombosis investigation system to obtain a rabbit ischemic stroke model

**DOI:** 10.1038/s41598-021-85348-6

**Published:** 2021-03-11

**Authors:** Yoonhee Kim, Yoon Bum Lee, Seung Kuk Bae, Sung Suk Oh, Jong-ryul Choi

**Affiliations:** 1 Medical Device Development Center, Daegu-Gyeongbuk Medical Innovation Foundation (DGMIF), Daegu, 41061 Republic of Korea; 2 Laboratory Animal Center, Daegu-Gyeongbuk Medical Innovation Foundation (DGMIF), Daegu, 41061 Republic of Korea; 3 Department of Biofibers and Biomaterials Science, Kyungpook National University, Daegu, 41566 Korea

**Keywords:** Biophotonics, Neuroscience, Optics and photonics, Brain injuries, Stroke

## Abstract

Photochemical thrombosis is a method for the induction of ischemic stroke in the cerebral cortex. It can generate localized ischemic infarcts in the desired region; therefore, it has been actively employed in establishing an ischemic stroke animal model and in vivo assays of diagnostic and therapeutic techniques for stroke. To establish a rabbit ischemic stroke model and overcome the shortcoming of previous studies that were difficult to build a standardized photothrombotic rabbit model, we developed a photochemical thrombosis induction system that can produce consistent brain damage on a specific area. To verify the generation of photothrombotic brain damage using the system, longitudinal magnetic resonance imaging, 2,3,5-triphenyltetrazolium chloride staining, and histological staining were applied. These analytical methods have a high correlation for ischemic infarction and are appropriate for analyzing photothrombotic brain damage in the rabbit brain. The results indicated that the photothrombosis induction system has a main advantage of being accurately controlled a targeted region of photothrombosis and can produce cerebral hemisphere lesions on the target region of the rabbit brain. In conjugation with brain atlas, it can induce photochemical ischemic stroke locally in the part of the brain that is responsible for a particular brain function and the system can be used to develop animal models with degraded specific functions. Also, the photochemical thrombosis induction system and a standardized rabbit ischemic stroke model that uses this system have the potential to be used for verifications of biomedical techniques for ischemic stroke at a preclinical stage in parallel with further performance improvements.

## Introduction

Stroke is a disease in which blood supply to certain parts of the brain is interrupted or bleeding occurs due to damage to cerebral blood vessels, resulting in damage to the brain tissue and nerves. According to a survey conducted by the World Health Organization (WHO)^1^, stroke is the second leading cause of death and the third-leading disease that causes disability. In a stroke report in the Republic of Korea (2018)^2^, 76% of all stroke cases are cerebral infarction (ischemic stroke), and the prevalence of strokes among people aged 75 or older is about 13 times higher than that in those aged 19–54 years. The risk of stroke is increasing due to the aging global population; thus, research on diagnosis and treatment techniques for stroke has been actively pursued. For instance, noninvasive stroke diagnosis techniques using high-speed X-ray angiography or magnetic resonance imaging (MRI) have been studied and employed in clinical sites^3–5^, and minimally invasive intervention procedures using cerebrovascular catheters have been adapted to stroke treatments^6,7^. Studies are also being actively conducted on early diagnosis reagents^8,9^ and therapeutic drugs^10,11^ for stroke.

As these studies and clinical applications become more active, the need for standardized stroke animal models and specific modalities to build them is increasing in response to validations of these technologies. Two important issues in setting up the stroke animal model are (1) how to build a stroke and (2) the type of animal to induce a stroke. The most common method for investigating an animal model of ischemic stroke, which accounts for three-quarters of stroke, is by injecting coagulated blood in a particular area or injecting endothelin-1 (ET-1) to close or mechanical vascular occlusion^12–14^. Mechanical occlusion requires considerable proficiency, and the modalities of cerebrovascular occlusion using coagulated blood or ET-1 have the disadvantage of not being able to precisely control the degree or locations of brain damage caused by artificial stroke. A photochemical thrombosis is a stroke animal model investigation modality that overcomes these shortcomings. Photochemical thrombosis forms a stroke and results in brain damage by injecting Rose Bengal, which forms blood clots within the blood vessels in response to a certain wavelength of light (~ 520 nm), and examining the light in a certain area of the brain that causes a stroke^15–18^. Although there is a limitation of photochemical vascular damage that can show physiological phenomena different from the actual clinical situation and progression, photochemical thrombosis is widely applied to the establishment of stroke-inducing animal models with localized brain damage and in the preclinical research of diagnostic and therapeutic techniques because it can precisely control the location and size of stroke-derived lesions by adjusting the location and light intensity. Furthermore, the type of animal depends on the purpose and function of the research. Mice and rats are generally employed in the advantage of being economical and able to efficiently conduct experiments under multiple conditions; however, they are difficult to connect directly to actual clinical trials due to their wide differences with the human brain^19^. However, large animals and primates are inefficient and difficult to study under multiple conditions. To overcome these limitations, studies have been conducted using animal models that investigate stroke in rabbits to develop efficient diagnostic and therapeutic techniques^20–22^. However, unlike the developments and applications of photochemical thrombosis-inducing systems on mice and rats^18,23–25^, a system that can investigate stroke rabbit models consistently and a platform that can verify brain damage by obtaining images noninvasively and longitudinally is scarce, and development is needed to investigate a quantitatively standardized stroke rabbit model using the well-established platform.

In this study, we developed a photochemical thrombosis investigation system to establish a rabbit ischemic stroke model and confirmed that this system can produce consistent brain damage to target areas of the rabbit brain. MRI was employed to analyze stroke-induced brain damage noninvasively, longitudinal imaging using T1- and T2-weighted images was performed to obtain both structural brain and infarction information, and artificial stroke and their transient changes were observed. In addition, 2,3,5-triphenyltetrazolium chloride (TTC) staining was applied to the sliced brain tissue to confirm stroke and measure the derived brain damage volume, and using hematoxylin and eosin (H&E) staining and Cresyl violet (Nissl) staining, we determined the severity of the morphological change of brain cells. Histological staining was also applied to confirm the correlation with MRI. Furthermore, the photochemical thrombosis investigation system and analytical techniques of stroke and it-induced brain damage can be employed efficiently in preclinical studies of diagnostic and therapeutic methods using stroke rabbit models.

## Materials and methods

### System configurations

**Figure 1:**
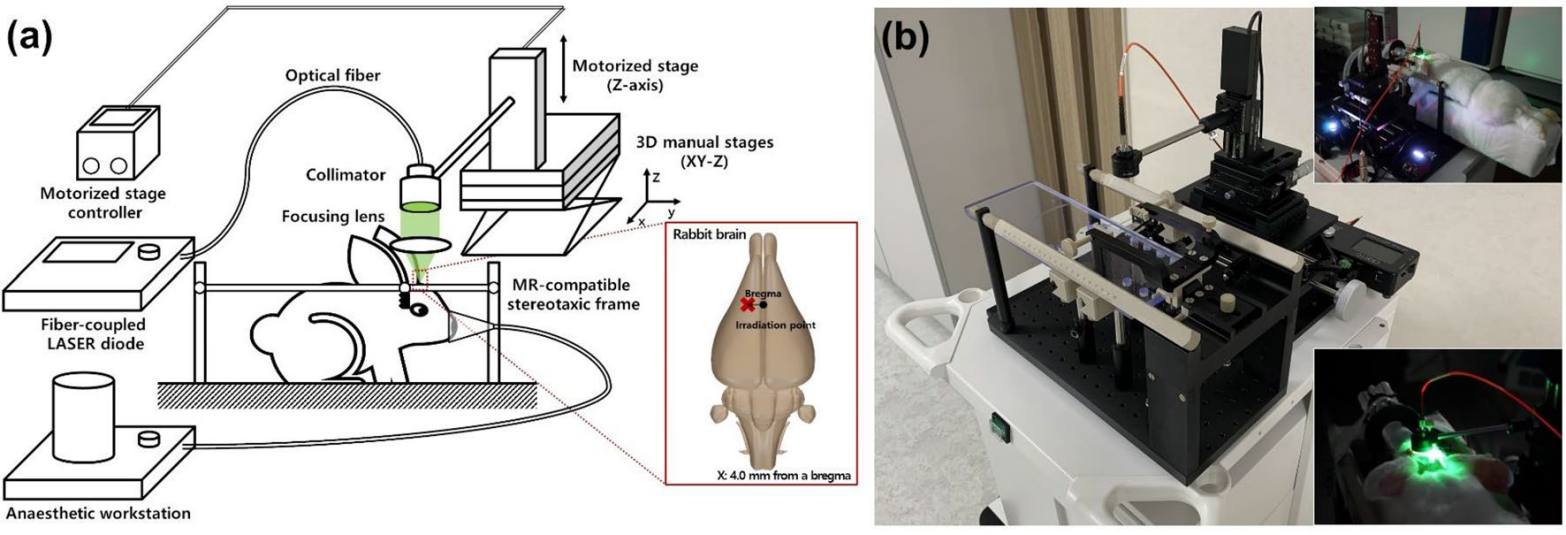
(**a**) Schematic of a photochemical thrombosis investigation system to establish the ischemic stroke middle animal (rabbit) model. The system consists of a stereotaxic frame for positioning an animal, a light irradiation part for inducing photochemical thrombosis in a specific area, and an irradiation probe-positioning part consisting of a three-dimensional manual stage and a motorized stage for controlling the lateral and axial positions accurately. Using the developed system, light for localized photochemical thrombosis was exposed at a distance of 4.0 mm in the X-axis from a bregma of a rabbit brain, which is an area corresponding to the motor cortex of the hind leg, as described in an inlet figure. (**b**) Image of an entire photochemical thrombosis investigation system built on a medical cart for mobility. The inlet figure indicates the system used in an experiment for establishing the photothrombotic rabbit stroke model.

A schematic of the photochemical thrombosis investigation system used to establish stroke rabbit models is shown in Fig. 1a. The system consisted of three parts: (1) a stereotaxic frame to position a rabbit, (2) a light irradiation part to induce photochemical thrombosis in a specific region of the rabbit brain, (3) and a precise positioning part including three-dimensional manual stages and a motorized stage to control lateral and axial positions accurately. The non-magnetic stereotaxic frame for middle animals (68915, RWD Life Science, Shenzhen, China) and ear bars designed for positioning rabbits were applied to the stereotaxic frame. Additionally, the base plate of the frame is modified to be installed on the optical breadboard to connect other components. In the light irradiation part, a single-channel fiber-coupled laser diode (LDFLS_520/060, Doric Lenses, Inc., Quebec, Canada) with a peak wavelength of 520 nm and a maximum optical power of 60 mW was used as a light source that reacts with Rose Bengal that photo-activating agent and causes photothrombotic occlusion of cerebral blood vessels. To deliver light to a specific region, a probe consisting of an optical fiber (M122L01, Thorlabs, Newton, NJ, United States), a fiber-coupled collimator (F240FC-532, *f* = 7.86 mm, Thorlabs), and a focusing lens (LA1027-ML, *f* = 35 mm, Thorlabs) were constructed and connected to the precise positioning parts. The three-dimensional stage was used to determine the brain damage region by precise positioning of the light irradiation probe consisted of two lateral stages (ACS-26, Namil Optical Instruments, Incheon, Republic of Korea), which can be moved in 10-micron units, and a manual lab jack (LJ-28, Namil Optical Instruments) and a motorized axial stage (MTS50/M-Z8, Thorlabs) additionally connected to it can be more accurately positioned by the controller (KDC101, Thorlabs). In the experiment, the light was irradiated with an output of 10 mW to cause a photothrombic stroke, which was pre-calibrated with a photodetector (S120C, Thorlabs) and an optical power meter (PM400, Thorlabs). An entire photochemical thrombosis investigation system built on a medical cart for mobility and the system employed in the establishment of a photothrombotic rabbit stroke model are shown in Fig. 1b.

### Animal preparation and photothrombosis-inducing procedures

Adult male New Zealand White rabbits weighing 2.3–2.4 kg and aged 12 weeks were used in this study. The animals were obtained from Samtaco (Osan, Republic of Korea). All experiments were approved by the Animal Experiment Ethics Committee of Daegu–Gyeongbuk Medical Innovation Foundation (approval number: DGMIF-19072501-01) and all procedures were conducted in accordance with the guideline. Also, the study was carried out in compliance with the ARRIVE (Animal research: reporting of in vivo experiments) guidelines (http://www.nc3rs.org.uk/page.asp?id=1357) and all efforts were made to minimize a number of animals and their pain. The rabbits were divided into the following groups: Group 1 (control, n = 1), Group 2 (photochemical thrombosis investigation with 10 mW laser irradiation and longitudinal observation for 3 days, n = 4), Group 3 (photothrombosis investigation with 10 mW laser irradiation and a histological assay a day later, n = 2). A total of seven rabbits were used, and all were allowed free access to food and water.

For inducing photothrombotic ischemia and confirming the establishment of the stroke, there were three experimental groups in the study. All groups of rabbits were anesthetized using an intramuscular (IM) injection of 15 mg/kg Zoletil (Zoletil 50 inj., Virbac Korea, Seoul, Republic of Korea) and 10 mg/kg Rompun (Rompun inj., Bayer Korea, Seoul, Republic of Korea) before the induction of photochemical thrombosis. After anesthesia, we shaved the hair on the rabbit’s head with clippers. Rabbits were exposed to 1.5% Isoflurane (Ifran Liq, Hana Pharm Co., Ltd., Seoul, Republic of Korea) in air and in oxygen with anesthesia Isoflurane vaporizer (Vetia, J&TEC Co., Ltd., Gimpo, Republic of Korea) and maintenance 1.2% during the surgical and laser irradiation procedure. A scalp incision was performed to expose the skull. For disinfection, povidone-iodine swabs (A17200231, Dongindang, Seoul, Republic of Korea) were used. Then, saline was frequently applied to protect the brain from overheating and skull craniotomy was performed with the left lateral to the bregma using an 8 × 8 mm hand-held cranial microdrill (78001, RWD Life Science). Also, the meninges were separated to expose the brain for irradiation. The target brain area was localized in the motor cortex of the left cerebral hemisphere. Rabbits were placed in the non-magnetic stereotaxic frame that established the photochemical thrombosis inducing system, and the brain was exposed.

For producing brain damage in the area associated with exercise in the right hind limb, the probe to irradiate the LASER for photothrombosis was laterally aligned with a distance of 4-mm in the X-axis relative to bregma (as an inlet figure in Fig. 1) by controlling manual translation stages. The height of the probe was also axially adjusted by the motorized stage in the photothrombosis induction system to maximize the transfer efficiency of the light. After the procedures to align the probe, Rose Bengal (330000, Sigma-Aldrich, St. Louis, MO, United States), which was diluted with saline to 10 mg/mL and protected from the light was injected through an ear vein at a dose rate of 80 mg/kg for 3 min, while the other light was shaded and the LASER light was exposed to generate photothrombosis. The total light exposure time to generate photothrombosis was 30 min and a peak wavelength of light was 520 nm. The control group underwent the same procedures of this experiment without surgical incision, skull craniotomy, and an injection of Rose Bengal. After the induction of photochemical thrombosis, HY-bond polycarboxylate bone cement (PN1160, Shofu, Inc., Kyoto, Japan) was placed on the skull surface, and the wound was closed with the 4/0 polydioxanone and 3/0 nylon sutures.

### Magnetic resonance imaging configurations

Multiple MR images were acquired every 24 h for three days immediately after the photochemical thrombosis investigation. From these MR images, the formation and progression of ischemic stroke area and the degree of brain tissue damage were measured. To increase the reproducibility of the position and region of the stroke, each region of interest (ROI) was determined so that the center of each slice is located on the crossline between two ends of the olfactory’s and vermis’s edges in the sagittal view of the rat brain. For observation of ischemic cerebral lesions, T1- (TR (Repetition time) = 600 ms, TE (Echo time) = 2.87 ms, FOV (field-of-view) = 128 × 128 mm^2^, matrix size = 256 × 256, number of slices = 25, slice thickness = 2.0 mm, averaging number = 3) and T2-weighted (TR = 9500 ms, TE = 99 ms, FOV = 128 × 128 mm^2^, matrix size = 256 × 256, number of slices = 25, slice thickness = 2.0 mm, averaging number = 3) MR imaging were acquired. For the high-resolution rabbit brain structure, the T1-weighted MPRAGE (Magnetization Prepared RApid Gradient Echo) image was also obtained with the following parameters: TR = 1310 ms, TE = 4.99 ms, FOV = 128 × 128 mm^2^, matrix size = 512 × 512, slice thickness = 0.5 mm, slice per slab = 112, number of slabs = 1, averaging number = 3. To measure areas and intensities in the photothrombotic brain damage from the MR images, the region of the cerebral lesion was extracted using semi-automatic segmentation, and an area and an averaged intensity with standard deviation in each selected region were estimated using numerical analysis software.

### Histological analysis

Conforming the establishment of the stroke in ex vivo brain slices and histological analysis were progressed after 72 h of photothrombosis induction. Experimental steps of histological analysis are as follows. At first, rabbits were anesthetized using an IM injection of 15 mg/kg Zoletil (Zoletil 50 inj., Virbac Korea) and 10 mg/kg Rompun (Rompun inj., Bayer Korea) before transcardial perfusion. Rabbits were transcardially perfused with ice-cold 1X phosphate-buffered saline (PBS; 7011044, Life Technologies Korea, Seoul, Republic of Korea) at 24 h and 72 h after inducing photochemical thrombosis. After perfusion, the rabbit brain was removed gently and rapidly. Coronal sections of the brain with a thickness of 4 mm were cut using a rabbit brain matrix (15026, Ted Pella, Inc., Redding, CA, United States). For infarcted area measurement, the brain sections were stained with 2% TTC (T8877-25G, Sigma-Aldrich) in PBS at 37 °C for 30 min. The solution was made immediately prior to use and protected from the light.

**Figure 2:**
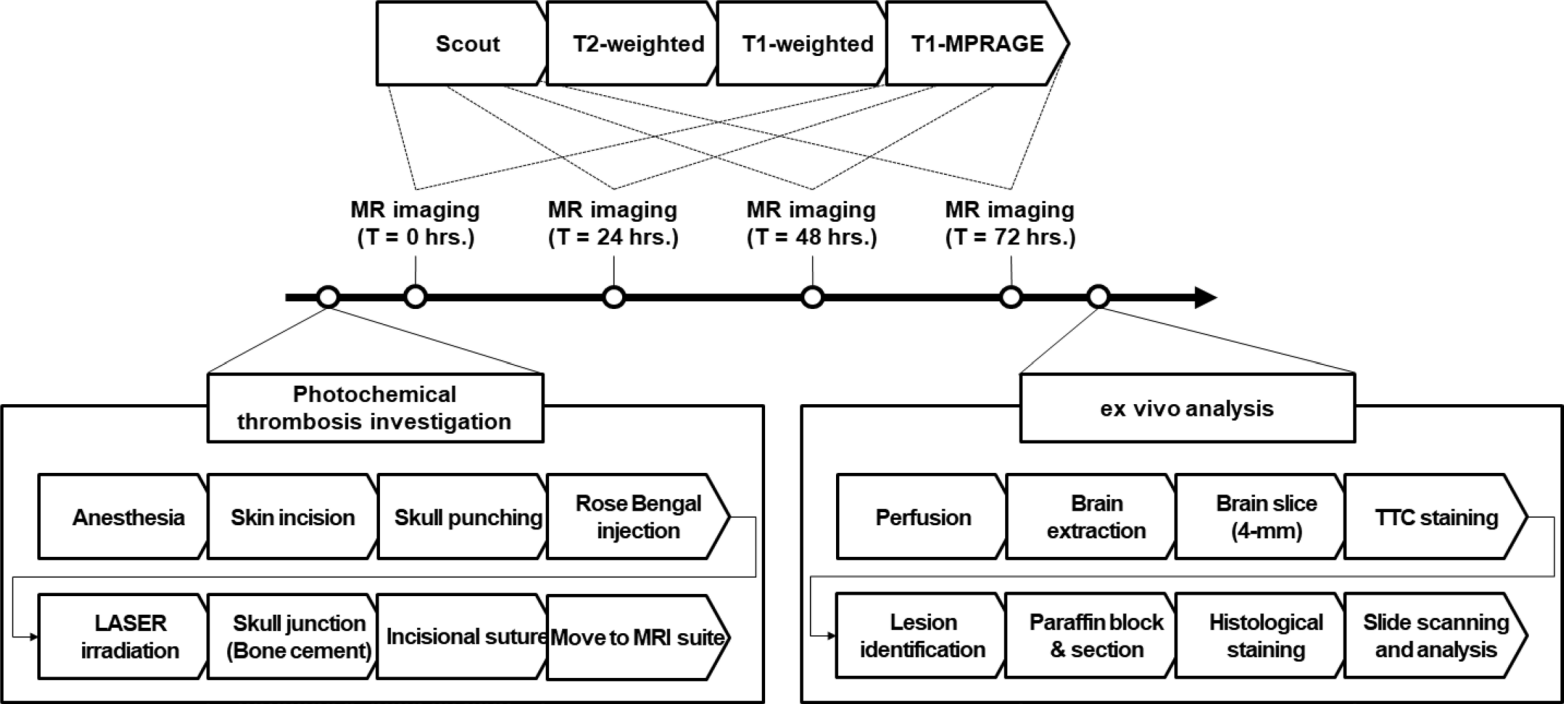
Schematic of procedures in ischemic stroke rabbit model establishments using the photochemical thrombosis investigation system. Longitudinal measurements were performed using magnetic resonance imaging (MRI) for three days to verify the system and noninvasively identify cerebral damages, and ex vivo analysis using multiple histological staining was performed for the cross-validation of photochemical thrombosis and it-derived brain damages after the longitudinal analysis by MRI.

For histological analysis, H&E and Cresyl violet staining was performed to assess the morphology of neurons and glial cells. The brain sections were fixed in 4% paraformaldehyde in PBS (M1176, Biostem, Suwon, Republic of Korea) for 24 h. The fixed brain was embedded in paraffin, and the paraffin block was cut to a thickness of 4 μm using a rotary microtome (RM2255, Leica Microsystems, Wetzler, Germany). Slides of rabbit brain paraffin sections were deparaffinized and hydrated using xylene (Duksan, Ansan, Republic of Korea) and graded alcohol series. To analyze the brain cells, the paraffin sections were stained with 0.1% Cresyl violet (C5042-10G, Sigma-Aldrich); to analyze the infarction area and cellular density, the sections were stained with H&E stain kit (H3502, Vector Laboratories, Inc., Burlingame, CA, United States). The sections were imaged using a Brightfield slide scanner (Zeiss Axio Scan.Z1, Carl Zeiss, Jena, Germany). The detailed procedures from animal preparations to histological assays are illustrated in Fig. 2.

## Results and discussions

### MR imaging of photothrombic stroke investigation in a rabbit brain

**Figure 3:**
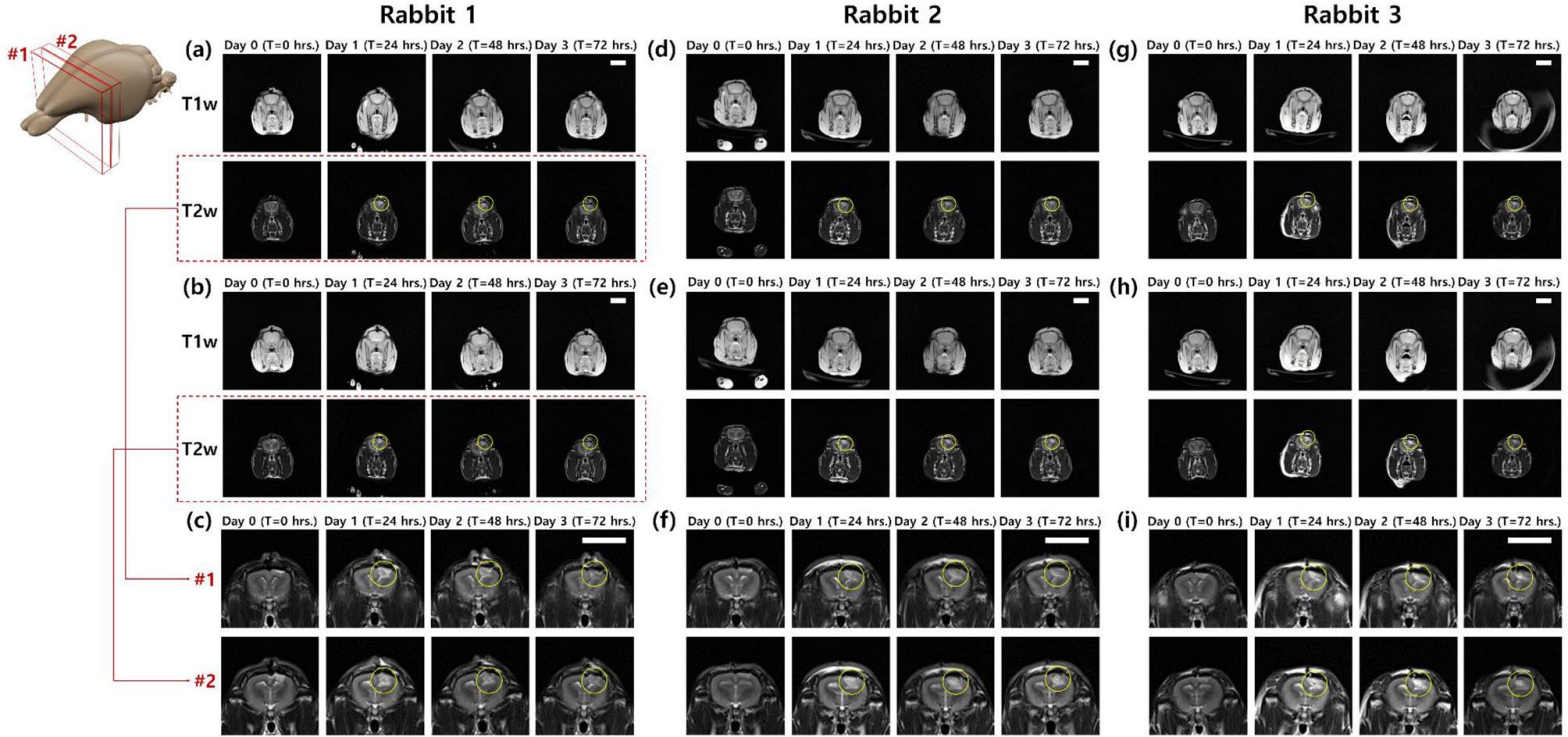
Transient (**a**) T1-, (**b**) T2-weighted, and (**c**) magnified T2-weighted magnetic resonance images of two brain slices (#1 and #2) of Rabbit 1 obtained immediately, 24, 48, and 72 h after the investigation of photochemical thrombosis. When identifying the area marked with a circle at T2-weighted images, the system well-formed localized ischemic stroke in the desired location and brain damage from the photothrombosis. Brain damages by photothrombosis-induced, localized ischemic stroke were confirmed in magnetic resonance brain images of (**d**–**f**) Rabbit 2 and (**g**–**i**) Rabbit 3. A scalebar in figures indicates 20 mm.

Longitudinal and noninvasive imaging of the rabbit brain with ischemic stroke induced using the photochemical thrombosis investigation system was performed by MRI, as shown in Fig. 3. Specifically, T1- and T2-weighted imaging was employed to observe brain damage induced by photothrombotic ischemic stroke in rabbits. T1-weighted imaging acquires spin–lattice relaxation by short TR and TE times to obtain images that have higher intensity in fat and brighten the area corresponding to the normal tissue^26–29^. However, T2-weighted imaging measured spin–spin relaxation by long TR and TE times for acquisitions of images that have higher intensity in more water content than normal tissue. Therefore, infarction caused by photothrombotic stroke appears brighter in T2-weighted images. As shown in Fig. 3, areas where photothrombotic stroke-induced lesions, with a higher intensity than normal brain tissues, emerged after 24 h of investigating photochemical thrombosis. Immediately after LASER irradiation to investigate photochemical thrombosis, brain damage by photothrombosis was not observed because there was a time interval when blood vessels in the area that received light were blocked and brain damage occurred. In the case of T1-weighted images, there was a slight difference in the intensity of the area with brain damage immediately after and after 24 h of photothrombosis investigation, but it was not as high as that in the T2-weighted images. When MR images corresponding to 24 h and 48 h after the photochemical thrombosis were identified, the sizes and intensities of brain damage were maintained in a measure, and MR images at 72 h after photothrombosis indicated a decrease in both size and intensity of the photothrombotic stroke-induced lesion. On the other hand, an area where high T2 intensities due to brain damages was not consistently identified over three days in MR images of a brain in a rabbit of a control group as described in Fig. S1. The transient trend of brain damage occurrence and recovery is consistent with the results of previous studies in the transient analysis of a photothrombotic rat stroke model^18,30^. Also, to determine whether the photochemical thrombosis investigation system can precisely control where light is irradiated and induce photothrombotic brain damage to the selected area, we conducted the induction of photothrombosis by irradiating the light at another location of 2.5 mm away on X-axis from the bregma. When comparing photothrombotic brain damage in MR images of a rabbit brain with light irradiations at a location of 2.5 mm (as described in Fig. S2) and 4.0 mm (as described in Fig. S3) away from the bregma, the center of the brain damage was formed closer to the bregma in the condition that the targeted location was 2.5 mm away from the bregma. We determined distances between a center of the brain damage and the bregma in magnetic resonance images. The measured distance was 4.00 mm when the targeted location of 4.0 mm was adjusted, and it was 2.66 mm when the targeted location was 2.5 mm. The result indicates that the developed photochemical thrombosis investigation system is capable of positioning the location of photothrombotic brain damage in sub-millimeters.

**Figure 4:**
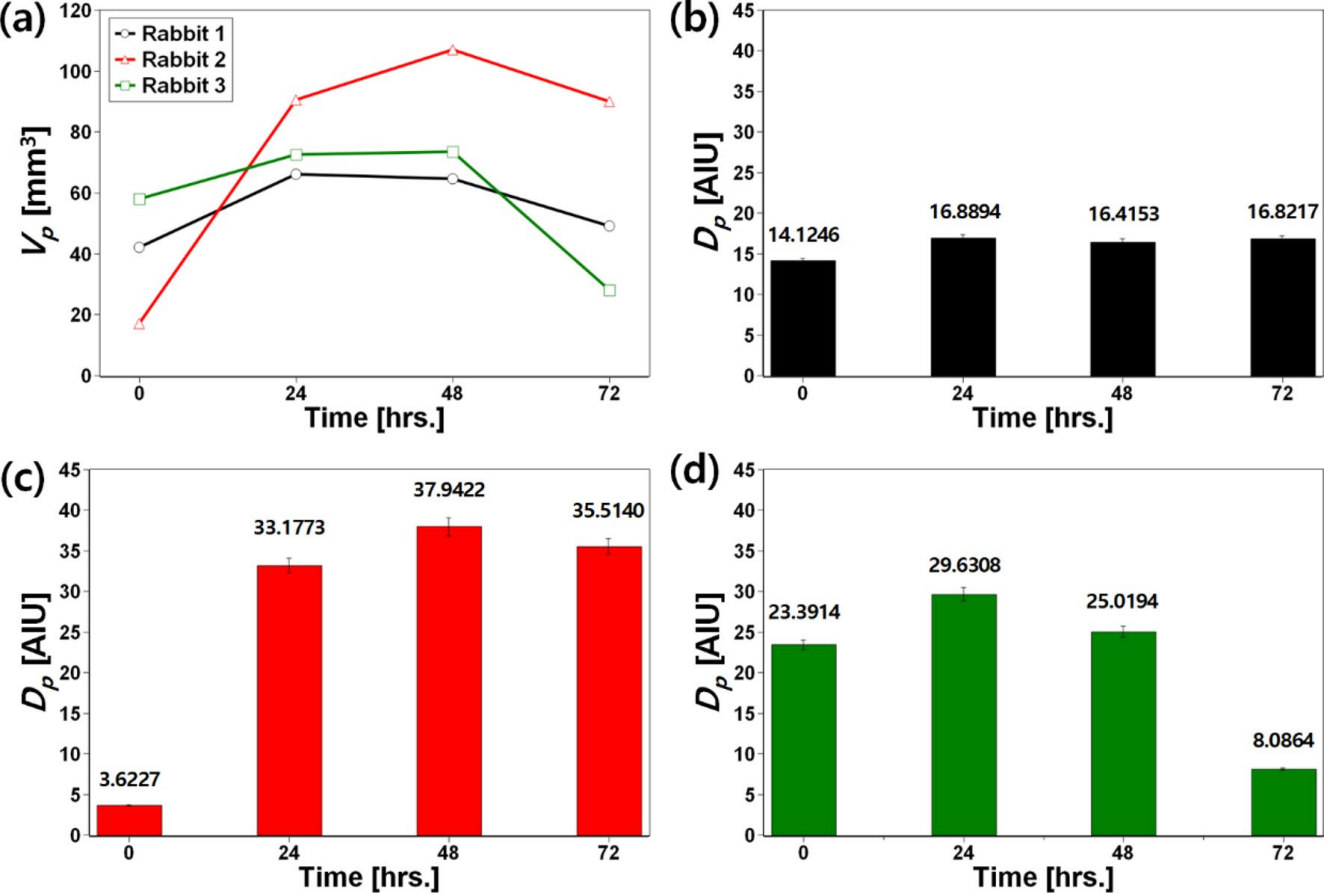
Quantitative and transient analysis of (**a**) volumes (${V}_{p}$) and (**b**–**d**) the degree of photothrombotic brain damage (${D}_{p}$) of three rabbit ischemic stroke models established. ${V}_{p}$ increases 24–48 h after the induction of photothrombosis and tends to decrease 72 h after the induction. In the identification of ${D}_{p}$, it was quantitatively confirmed that the brain damage caused by photothrombosis was greater after 24 h, which is correlated to the result of the qualitative analysis on T1- and T2-weighted MR images described in Fig. 3.

Figure 4 shows the quantitative data of volumes and intensities in damaged brain tissues segmented from T2-weighted MR images of three photothrombotic rabbit stroke models. To explain the quantitative analysis method of transient changes of brain damage in detail, the regions where photothrombotic brain damage occurred were extracted by a semi-automatic segmentation algorithm for each photothrombosis rabbit model and at each temporal point, and the volume of the extracted region (${V}_{p}$) was measured. In addition, the degree of photothrombotic brain damage (${D}_{p}$) was estimated as

$$\begin{aligned} D_{p} & = ~\iiint {\left( {T2\;intensity} \right)dV|_{{Photothrombotic\;lesion}} \times \left( {Unit\;voxel\;volume} \right)} \\ & = \;\left( {Average\;T2\;intensity} \right)|_{{Phothtrombotic\;lesion}} \times {\text{}}V_{p} \\ \end{aligned}$$

as an indicator for quantitative comparison of brain damage in longitudinal T2-weighted MR images. As shown in Fig. 4a, although there are individual differences, ${V}_{p}$ increases 24–48 h after the induction of photothrombosis and tends to decrease 72 h after it. It is correlated with the trend of inducing and recovering photothrombotic brain damage from prior studies on establishing photothrombosis-based ischemic stroke small animal models^18,30^. In the comparison of *D*_*p*_ (Fig. 4b–d), it was commonly confirmed that *D*_*p*_ in each photothrombotic rabbit stroke model increased after 24 h of photothrombosis (Rabbit 1: 14.1246 → 16.8894; Rabbit 2: 3.6227 → 33.1773; and Rabbit 3: 23.3914 → 29.6308), although there was a deviation. This means that the brain damage caused by photothrombosis was greater after 24 h, which is correlated with the results of the qualitative analysis on T1- and T2-weighted MR images, as shown in Fig. 3.

### Comparative verification using histological assays of ex vivo brain slices

**Figure 5:**
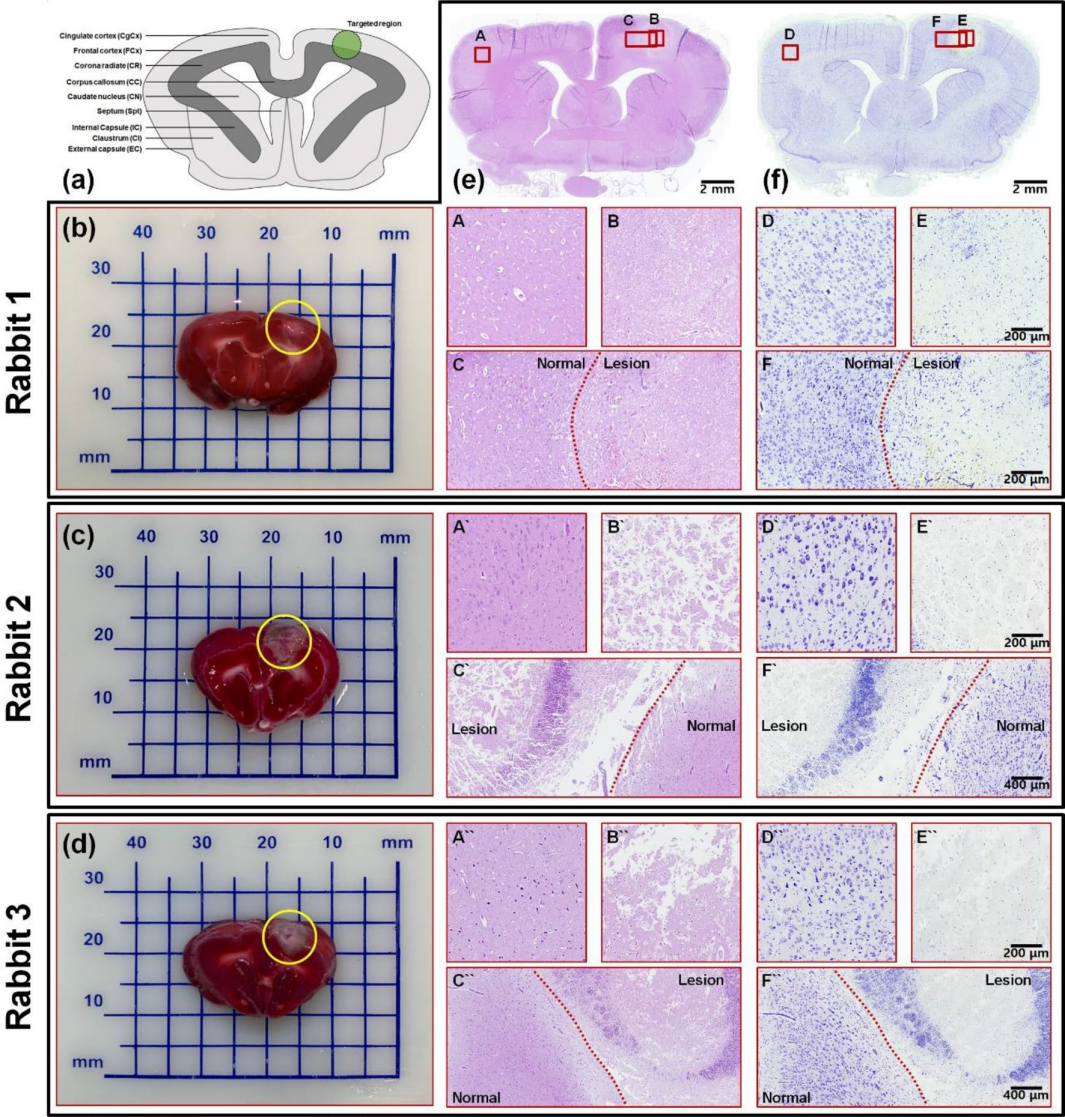
Histological analysis of the rabbit brain with a cerebral lesion 72 h after the photothrombotic ischemic stroke. (**a**) Schematic of the rabbit brain atlas indicating the localized photothrombotic ischemic stroke-investigating target region. (**b**–**d**) Photographs of the 4-mm brain slice stained using 2,3,5-triphenyltetrazolium chloride (TTC) to determine the location and volume of the photothrombotic brain damage in three rabbits. (**e**) H&E (hematoxylin and eosin) staining (**f**) and Cresyl violet (Nissl) staining brain tissue sections including the cerebral lesion caused by the photochemical thrombosis for rabbit brain described in (**b**). H&E- and Nissl staining images of the magnified area marked as a red box of tissue without lesion (A and D), where photothrombotic brain damage occurred (B and E) and at a boundary between the normal and damaged regions (C and F) are described below to compare the density of cellular components (nuclei, extracellular matrix, and cytoplasm) and neurons. H&E and Nissl staining images of tissues without lesion, where photothrombotic brain damage occurred and at a boundary between the normal and damaged regions corresponding to rabbit brains in (c) (A′ to F′) and (**d**) (A″ to F″) are described on the right side of each figure. H&E staining images of photothrombotic ischemic lesions (A, A′, and A″) of three rabbits had a lower density of cellular components than contralateral normal brain tissue (B, B′, and B″) due to the presence of vacuolation and shrinkage of neurons. Likewise, Nissl staining images of the ischemic lesion (D, D′, and D″) had a lower density of neuronal cells than the normal brain tissue (E, E′, and E″). In the Nissl staining image of lesions, nuclear shrinkage and pyknosis were observed.

To confirm the occurrence, size, and severity of brain damage induced using the photothrombotic ischemic stroke investigating system, we performed three staining methods to evaluate the ischemic lesion as shown in Fig. 5. First, TTC staining was applied to demonstrate brain tissue viability. This method is one of the most common staining methods for distinguishing the enzymatically dysfunctional cells from normal cells. In ischemic brain tissue, a lack of TTC represents the damaged cells and non-viable cells metabolic state. The TTC-negative (colorless) region indicates the infarcted region and mitochondrial dysfunction state. In viable brain tissue, the cells produce the dehydrogenase through cellular respiration; therefore, the TTC solution reacts with the dehydrogenase and the brain tissue changes to red. TTC is a direct indicator of ischemic lesions and damaged cell volume. MRI and TTC staining are commonly used together to evaluate brain damage, and both methods ensure precise measurement of the ischemic stroke area. We performed TTC staining of brain sections 72 h after inducing photothrombotic ischemia and observed the colorless region on the laser irradiation target area. Additionally, we found a high correlation between the MRI scan image and TTC staining.

For the histological assay, we used H&E staining of the brain sections. The inlet figure of B in Fig. 5 shows the ischemic core region and that of C in Fig. 5 shows the intact region boundary morphology. We observed a significant decrease in cells of the infarct area and confirmed similar morphological changes in other groups. To distinguish neurons glia from other cells, we performed Nissl staining. We observed nuclear shrinkage and pyknosis, which are characterized as ischemic neurons^31,32^. In the H&E staining image shown in Fig. 5 (C′ and C″) around the infarct boundary zone, there are darkly stained pyknotic nuclei without the cytoplasm. Nissl staining tissues in Fig. 5 (E, E′, and E″) show the ischemic region’s degenerating neurons that appear pale and demonstrate that our photothrombotic ischemic inducing system produced the brain stroke. This result is caused by photochemical thrombosis, which is correlated with the histological analysis of a small animal model of ischemic stroke induced by MCAO^33,34^.

To semi-quantitatively determine whether photothrombosis-derived brain damage affects tissue components including neuronal cells and extracellular matrices, H&E stained brain tissue images (as shown in inlet images of Fig. 5) with a size of 774,400 μm^2^ for photothrombotic cerebral lesion (A, A′, and A″) and contralateral normal brain tissue (B, B′, and B″) were segmented, and a tissue component coverage ratio in each H&E staining image was calculated by grayscale intensity thresholding. The average and deviation of the tissue component coverage ratio in the photothrombotic lesion for the three rabbit stroke models were 51.784 ± 14.287%. In the normal brain tissue, the average and deviation of the coverage ratio were 94.855 ± 3.507%. This means that photothrombotic ischemic stroke induced using the photochemical thrombosis investigation system can damage the cells and extracellular matrices in localized and selective regions.

For the semi-quantitative comparison of a number of cells stained by Cresyl Violet, an index was determined by the mean of the intensity (grayscale value) in Nissl staining images of neuronal cells and estimated for each segmented image in Fig. 5. The result inferred that the number of Nissl-stained cells in the photothrombotic lesion (D, D′, and D″) was decreased by about 37.88 ± 11.09% compared to the amount of the stained cells in the contralateral (normal) tissue (E, E′, and E″).

### Discussion in improvements and applications

The photochemical thrombosis investigation system established in this study is meaningful in forming ischemic stroke, a consequent localized brain damage in a selected location of the rabbit brain, and in producing a uniform rabbit ischemic stroke model. In previous studies using a photothrombotic rabbit model^22,35,36^, a position of light irradiations in photothrombosis was not accurately controlled and light was exposed in a roughly selected area. For this reason, establishments of standardized photothrombotic animal model is difficult by photothrombosis investigation modalities in previous studies. This study focused on improving the shortcoming of previous studies by developing the photothrombosis investigation system to control precisely where light for photothrombosis is irradiated using a stereotaxic frame and a multi-axis translation stages, and suggesting that the system can be used to induce localized cerebral damage and build photothrombotic rabbit models. The system, which was combined with a laser irradiation probe connected to accurate multi-dimensional translation stages and a modified stereotaxic frame for middle animals, demonstrated sufficient potential to determine the precise irradiation position around a bregma in the brain and to generate photochemical thrombosis. In conjugation with brain atlas, it can induce photochemical ischemic stroke locally in the part of the brain that is responsible for a particular brain function and the system can be used to develop animal models with degraded specific functions.

One of the additional applicable methods to establish a standardized photothrombotic stroke animal model is the feedback of laser irradiation optical power to provide a more consistent degree of photothrombotic stroke-induced brain damage. Specifically, the application of optical components to a fiber-coupled laser irradiation probe that partially separates the output of a laser, such as a pellicle beam splitter^24^ or a bifurcated optical fiber, and the feedback that regulates input currents applied to the light source from the measured optical power of partially separated laser irradiation can prevent changes in laser irradiation and unscheduled brain damage caused by sudden fluctuations. The modality of laser irradiation, which maintains a constant axial distance from brain tissues by integrating a noncontact distance sensor based on light^37,38^ or ultrasound^39^ to the tip of the laser irradiation probe, can also help create a consistent depth and degree of photothrombotic brain damage and establish a standardized ischemic stroke animal model.

Unlike the method in which large blood vessels are occluded to cause ischemic stroke, photochemical thrombosis is a modality that forms blood clots and induces ischemic stroke by damaging endothelial cell membranes in blood vessels of the area where green light is irradiated and interacts with Rose Bengal^16,40^. For this reason, time-of-flight MR angiography, which performs imaging and analysis of arterial blood vessels in an artery occlusion-based stroke animal model, is not appropriate for the verification and analysis of vascular information in photochemical thrombosis, which causes localized stroke by blocking small vessels in the irradiated region. For cerebrovascular information in localized photothrombotic stroke, high-resolution mesoscale imaging tools are required. Several studies suggested the possibility that laser speckle vascular imaging can provide information on vascular changes and occlusion of surface capillaries for photothrombotic stroke^30,41–43^. Photoacoustic vascular imaging, which has a larger depth-of-imaging than laser speckle imaging, can also be applied as an analytical tool for capillary changes and occlusion during and after the investigation of photochemical thrombosis^44–46^.

To improve the longitudinal analyses of photothrombotic cerebral lesions by MR imaging, there are two main application directions: one is to apply imaging sequences to acquire functional brain images. Diffusion-weighted imaging (DTI)^47,48^ and MR-based blood oxygenation level-dependent (BOLD) imaging^49,50^ can provide functional information during and after the investigation of photochemical thrombosis. The other direction is to develop and employ imaging protocols to observe differences in intensity due to ischemic stroke-induced brain damage more clearly than T2-weighted MR imaging. Dynamic contrast-enhanced (DCE) MR imaging^51,52^ and susceptibility-weighted imaging (SWI)^53,54^, which extracts susceptibility maps from information of magnitude and phase in MR images, are techniques that can provide highly sensitive MR images from stroke and it-derived cerebral lesions, and the application of these imaging sequences can obtain more detailed information on the occurrence and recovery of brain damage. The employment of coils designed and manufactured tailored to middle animals is also a method to improve MR signal acquisition sensitivity^55,56^.

In this study, we applied MRI and histological staining to confirm the correlation of ischemic damage induced using the photothrombotic ischemic stroke investigating system. The results of this experiment showed that the photochemical thrombosis inducing system produced ischemic damage in the laser irradiation target area. The damage volume of the target region is a significantly important parameter for the assessment of induced ischemic damage severity and our system’s reliability.

Our previous study^18^ confirmed the enzymatically dysfunctional cells not only in neurons but also in all types of cells. To analyze and confirm the damage at the cellular level, we used multiple histological techniques such as H&E and Nissl staining for detailed biological assessment. For a more accurate analysis of neuron and glial cells, further staining methods can be employed. Immunohistochemical staining can detect neurons and other glial cells, respectively. NeuN is a marker for mature neurons, anti-glial fibrillary acidic protein (GFAP) indicates the infarcted boundary between the ischemic and normal regions, and CD68 is a marker for macrophage and microglia cells^30,57,58^. The anti-von Willebrand factor (vWF) can detect endothelial cells, and anti-α-smooth muscle actin (α-SMA) is a marker of vascular smooth muscle cells^30,59,60^. In addition, fluorescent anionic dyes such as Fluoro-Jade^57,61,62^ specifically stains the soma of the neuron and binds to degenerating neurons.

The established photochemical thrombosis investigation system can be applied to preclinical studies of advanced biomedical techniques that require precise light irradiation. Among them, photodynamic therapy is a representative applicable biomedical technique. Recently, there have been studies on photodynamic therapy, which uses Rose Bengal to induce death in cancer cells to treat tumor diseases^63–66^. Due to the similarity with the principle of photo-activation of Rose Bengal by irradiation, the established system can be applied to preclinical studies in photodynamic therapy using Rose Bengal, and the system has the potential to be employed in investigating optimized treatment protocols according to laser irradiation output and locations in preclinical assays. In addition, a standardized rabbit photothrombotic stroke model, which was established in this study and techniques to analyze brain damage derived from photothrombosis, have the potential to be employed in the development and verification of medical devices. For instance, a transcranial electrical stimulator^67–71^, and drugs^72,73^ to treat acute ischemic stroke.

## Concluding remarks

In this study, a photochemical thrombosis investigation system using multi-dimensional translation stages and a middle animal stereotaxic frame was established for the development of a standardized photothrombotic ischemic stroke rabbit model with brain damage in a target region for overcoming the shortcoming of previous studies that were difficult to build a standardized photothrombotic middle animal model. Through longitudinal T1- and T2-weighted MR imaging and histological assays such as TTC, H&E, and Cresyl Violet staining. It was confirmed that the cerebral lesion induced by photochemical thrombosis was formed in the specific target position using the developed system. The results of this study indicated that the photochemical thrombosis investigation system and standardized photothrombotic stroke rabbit model built using it have potential for application in the verification of therapeutic techniques for ischemic stroke at a preclinical stage and the development of various medical devices based on biophotonics in parallel with further performance improvements.

## Supplementary Information

Supplementary Information
