# Supplementary material for: Development of a photochemical thrombosis investigation system to obtain a rabbit ischemic stroke model
Source: Sci Rep. 2021 Mar 11;11:5787. doi: 10.1038/s41598-021-85348-6 (PMC7970995; doi:10.1038/s41598-021-85348-6)
Supplement: Supplementary file 1 — Supplementary Information [file 41598_2021_85348_MOESM1_ESM.docx]

**Supplementary Information**

**Development of a photochemical thrombosis investigation system to obtain a rabbit ischemic stroke model**

**Yoonhee Kim^1^, Yoon Bum Lee^2^, Seung Kuk Bae^3^,
Sung Suk Oh^1,*^, Jong-ryul Choi^1,*^**

1 Medical Device Development Center, Daegu-Gyeongbuk Medical Innovation Foundation (DGMIF), Daegu, 41061, Republic of Korea

2 Laboratory Animal Center, Daegu-Gyeongbuk Medical Innovation Foundation (DGMIF), Daegu, 41061, Republic of Korea

3 Department of Biofibers and Biomaterials Science, Kyungpook National University, Daegu, 41566, Korea

* Correspondence: S. S. Oh ([ssoh@dgmif.re.kr](mailto:ssoh@dgmif.re.kr); Tel: +82-53-790-5615) and J. Choi ([jongryul32@dgmif.re.kr](mailto:jongryul32@dgmif.re.kr); Tel: +82-53-790-5614)

*Keywords: Photochemical thrombosis; Ischemic stroke; Magnetic resonance imaging;*

*Histological analysis; Brain damage; Longitudinal brain imaging;*

*Rabbit ischemic stroke model;*


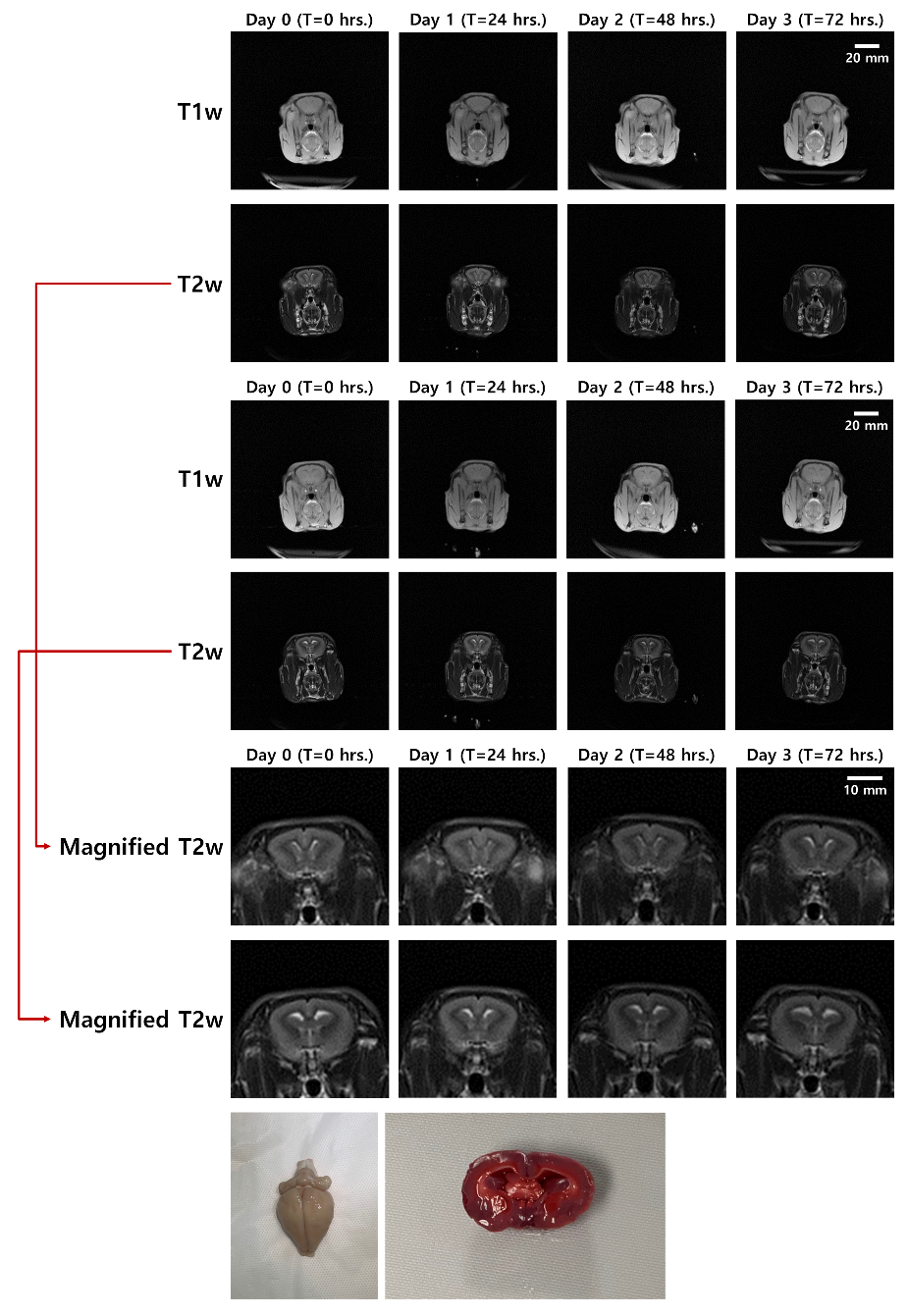


**Figure S1**. Transient T1-, T2-weighted and magnified T2-weighted magnetic resonance images of two brain slices of control rabbit obtained immediately, 24, 48, and 72 hours. We confirmed that the significant intensity differences corresponding to brain damage in the T2-weighted image of photothrombosis-induced rabbit brain (**Fig. 3** in the manuscript) is not visible in the brain image of the control rabbit. Similarly, photographic images of an extracted brain and a TTC-stained brain section, which are described in the bottom images of **Fig. S1**, indicated no significant signs of brain damages.


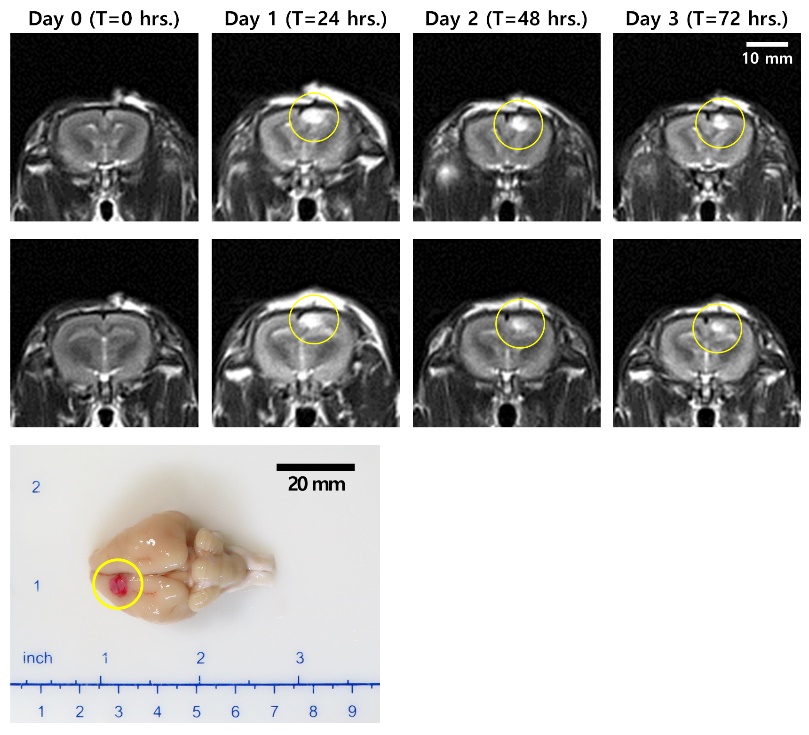


**Figure S2**. Magnified T2-weighted magnetic resonance images and photographic image of a rabbit brain with cerebral lesion to be established by photothrombosis with light irradiations at a location of 2.5 mm away on X-axis from the bregma.


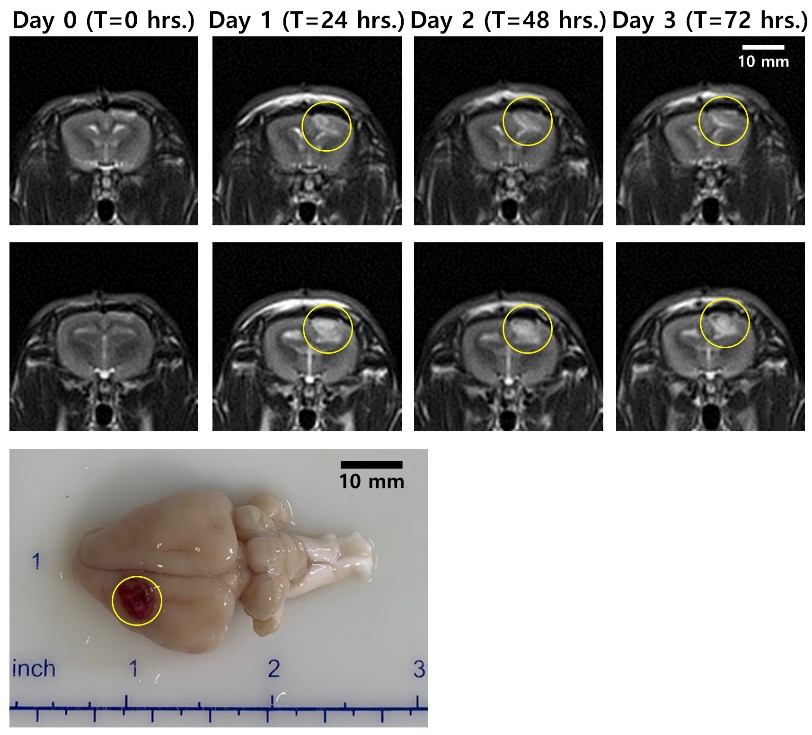


**Figure S3**. Magnified T2-weighted magnetic resonance images and photographic image of a rabbit brain with cerebral lesion to be established by photothrombosis with light irradiations at a location of 4.0 mm away on X-axis from the bregma.
